# Application of machine learning methodology to assess the performance of DIABETIMSS program for patients with type 2 diabetes in family medicine clinics in Mexico

**DOI:** 10.1186/s12911-019-0950-5

**Published:** 2019-11-12

**Authors:** Yue You, Svetlana V. Doubova, Diana Pinto-Masis, Ricardo Pérez-Cuevas, Víctor Hugo Borja-Aburto, Alan Hubbard

**Affiliations:** 1 Division of Epidemiology and Biostatistics, School of Public Health, University of California, Berkeley, USA; 2 Epidemiology and Health Services Research Unit, CMN Siglo XXI, Mexican Institute of Social Security, Av. Cuauhtemoc 330, Col. Doctores, Mexico City, Mexico; 3 Interamerican Development Bank, 1300 New York Ave NW, Washington DC, 20577E USA; 4 Interamerican Development Bank, 6 Montrose Road, Kingston 6, Jamaica; 5 Direccion de Prestaciones Medicas, Instituto Mexicano del Seguro Social, Mexico City, Mexico; 6 Division of Epidemiology and Biostatistics, School of Public Health, University of California, Berkeley, USA

**Keywords:** Machine learning methodology, Diabetes program, Family medicine clinics, Mexico

## Abstract

**Background:**

The study aimed to assess the performance of a multidisciplinary-team diabetes care program called DIABETIMSS on glycemic control of type 2 diabetes (T2D) patients, by using available observational patient data and machine-learning-based targeted learning methods.

**Methods:**

We analyzed electronic health records and laboratory databases from the year 2012 to 2016 of T2D patients from six family medicine clinics (FMCs) delivering the DIABETIMSS program, and five FMCs providing routine care. All FMCs belong to the Mexican Institute of Social Security and are in Mexico City and the State of Mexico. The primary outcome was glycemic control. The study covariates included: patient sex, age, anthropometric data, history of glycemic control, diabetic complications and comorbidity. We measured the effects of DIABETIMSS program through 1) simple unadjusted mean differences; 2) adjusted via standard logistic regression and 3) adjusted via targeted machine learning. We treated the data as a serial cross-sectional study, conducted a standard principal components analysis to explore the distribution of covariates among clinics, and performed regression tree on data transformed to use the prediction model to identify patient sub-groups in whom the program was most successful. To explore the robustness of the machine learning approaches, we conducted a set of simulations and the sensitivity analysis with process-of-care indicators as possible confounders.

**Results:**

The study included 78,894 T2D patients, from which 37,767patients received care through DIABETIMSS. The impact of DIABETIMSS ranged, among clinics, from 2 to 8% improvement in glycemic control, with an overall (pooled) estimate of 5% improvement. T2D patients with fewer complications have more significant benefit from DIABETIMSS than those with more complications. At the FMC’s delivering the conventional model the predicted impacts were like what was observed empirically in the DIABETIMSS clinics. The sensitivity analysis did not change the overall estimate average across clinics.

**Conclusions:**

DIABETIMSS program had a small, but significant increase in glycemic control. The use of machine learning methods yields both population-level effects and pinpoints the sub-groups of patients the program benefits the most. These methods exploit the potential of routine observational patient data within complex healthcare systems to inform decision-makers.

## Background

In Mexico, type 2 diabetes (T2D) is a major public health concern. The prevalence of this condition is above 9.4% in the adult population and increasing [1]. T2D is a chronic disease characterized by a progressive loss of β-cell insulin secretion and frequent insulin resistance [2]. In poorly controlled patients, the chronic hyperglycemia causes damage of multiple organ systems and development of micro- and macrovascular complications. The manifestations of microvascular complications are nephropathy, retinopathy and neuropathy. Macrovascular complications are coronary artery disease, peripheral arterial disease, and stroke. These complications are accountable for most of the morbidity, hospitalizations, and deaths that occur in patients with diabetes mellitus [3, 4]. A recent meta-analysis of 28 randomized trials that included 34,912 T2D patients found that targeting intensive glycemic control (HbA1C < 7%) reduces the risk of microvascular complications, compared with conventional glycemic control; yet, it also increases the risk of hypoglycemia and did not show significant differences for all-cause and cardiovascular mortality [5].

The Mexican Institute of Social Security (Spanish acronym IMSS), is the most extensive health system in Mexico with nearly 65 million affiliates provides care to approximately 3.8 million T2D adult patients. The growing demand and healthcare requirements of T2D pose a heavy burden for family medicine clinics (FMCs), the frontline of IMSS healthcare. T2D patients are the second cause of consultation at FMCs, and those with acute and chronic complications, including comorbidities (i.e., hypertension) are among the top ambulatory and emergency consultations and hospital admissions [6]. Furthermore, T2D has substantial economic consequences, since in 2016, diabetes expenditures alone accounted for US$2.5 billion [7].

T2D is a complex chronic condition that requires multidisciplinary healthcare and strict patient’s adherence to reduce the risk of acute and chronic complications. The primary goal of T2D treatment is to reach glucose control (glycated hemoglobin -HbA1C- below 7%). Conventionally, IMSS FMC consultations and follow-ups for T2D have been provided by a family doctor including physical examination, laboratory tests (i.e., blood glucose) prescription of treatment and self-care counseling. The family doctor refers patients to the dietitian, social worker, ophthalmologist or other specialists for a consultation, but the frequency of referrals and waiting time to receive multidisciplinary care might last several weeks or months due to the limited supply of these specialists and the increasing demand of patients with T2D. An analysis of the electronic health records of 25,130 T2D patients found that only 13% were referred to an ophthalmologist, 3.9% received nutritional counseling, and 23% had HbA1c < 7% (or plasma glucose ≤130 mg/dl) [8]. Though there are specific clinical guidelines for T2D treatment, care is irregular and uncoordinated [9]. Evaluations of patient outcomes of FMCs at IMSS revealed less than 30% of T2D patients achieved HbA1c below 7% [10–12].

The need to improve health outcomes of T2D patients prompted IMSS to design and launch the DIABETIMSS program in 2008. DIABETIMSS is a comprehensive model of care that fulfills the Chronic Care Model attributes [13, 14]. The building block of DIABETIMSS is a multidisciplinary team (medical doctor, nurse, psychologist, dietitian, dentist, and social worker) that delivers coordinated and comprehensive healthcare. In addition to regular consultations with the team, T2D patients receive individual, family and group education on self-care and prevention of complications. Only T2D patients with less than 10 years after diagnosis and without severe chronic complications are eligible to enter DIABETIMSS. The primary goal of DIABETIMSS focuses on improving patient’s self-care and achieving glycemic and metabolic control (reducing high blood pressure, cholesterol levels, and excess body fat, among others). Ultimately, DIABETIMSS care is expected to avert acute complications, reduce demand for emergency services and hospitalizations and delay the progression of organ damage.

The program has expanded gradually. Currently, ~ 91,000 patients attend 136 DIABETIMSS program modules distributed throughout the country. DIABETIMSS introduced healthcare delivery changes for which an effectiveness evaluation is worthwhile. Previous evaluations of DIABETIMSS reported improvements in patient self-care and reductions in blood glucose levels. However, small samples and lack of a control group limit drawing robust conclusions [15–17].. In fact, in complex health systems, such as IMSS, it might not be possible to evaluate a new program by design, as it can be impractical to randomize the initiation of the program across different clinics for logistic and organizational reasons; therefore, to evaluate the impact of a program one often must rely on observational data.

A new trend of statistical approaches such as machine learning methods (e.g., the approaches used herein: Targeted Learning [18, 19] and Super Learning [20]) have been developed to use routine health data (e.g., electronic health records) to adjust for confounding and produce robust results that estimate parameters, such as the average treatment effect (ATE). The use of machine learning to estimate the data-generating distribution avoids assumptions implicit in standard parametric methods, mainly when there are many factors that can influence the outcome of interest. The ensemble learning method used in this paper is Super Learning [20], which builds an ensemble learner by choosing a weighted combination of algorithms (candidate learners) to optimize the predictive performance using (V-fold) multiple cross-validations. Some of these algorithms could be parametric models, while others could be machine learning algorithms. This ensemble learner is proved to achieve oracle inequality, which means that it is optimal in most typical situations where theory does not guide on which algorithm will be most successful for a given problem. This method allows straightforward generalizations that can accommodate complex data structures, including missing and censored data. If the relevant variables have been measured (such as all confounders of the intervention), this method allows for meaningful use of the routine observational big data to obtain results with a reduced statistical bias on the health programs effects useful for decision-makers, particularly in resource-limited settings where large scale trials are not possible.

The availability of the new statistical techniques provides a unique opportunity to ascertain how the combination of routine clinical data and statistical algorithms serve to evaluate the performance of the program. Therefore, the objective of the study was to assess the performance of DIABETIMSS on glycemic control of T2D patients, using available observational patient data and machine-learning-based Targeted Learning (TMLE) methods.

## Methods

We performed secondary data analysis of the electronic health record and laboratory databases from eleven IMSS’ FMCs located in Mexico City and the State of Mexico, for the period 2012 to 2016. The study included six clinics with DIABETIMSS program and five clinics that provided the conventional model of care (Table 1). The FMCs were selected by convenience and comprised clinics that had complete laboratory databases for the period analyzed.

**Table 1:** Characteristics of family medicine clinics and number of diabetic patients included in the analysis

Clinic Mask *N* = 11	Group	Consulting Rooms^a^ (n)	People affiliated (n)	People with type 2 diabetes with at least one medical consultation during the analyzed year					People referred and attend to DIABETIMSS program at least once				
				2012	2013	2014	2015	2016	2012	2013	2014	2015	2016
				(n)	(n)	(n)	(n)	(n)	(n)	(n)	(n)	(n)	(n)
A	Diabetimss	16	76800	3602	3476	3636	4333	4315	290	249	198	344	529
B	Diabetimss	21	100800	6098	6237	6372	6603	6785	613	1755	2223	1916	1510
C	Diabetimss	25	120000	8960	9366	9749	10183	10436	2119	1818	1566	2000	1918
D	Diabetimss	12	57600	4009	4125	4194	4334	4480	534	1274	1752	1777	1588
E	Diabetimss	10	48000	2217	2443	2560	2684	2846	512	531	496	423	407
F	Diabetimss	10	48000	2440	2691	2964	3049	3238	334	390	418	363	144
G	Conventional	29	139200	7664	7862	8068	8246	8579	0	0	0	0	0
H	Conventional	30	144000	8169	8324	8338	9081	9333	0	0	0	0	0
I	Conventional	12	57600	3005	3349	3441	3611	3950	0	0	0	0	0
J	Conventional	10	48000	2867	2988	3040	3260	3247	0	0	0	0	0
K	Conventional	10	48000	2475	2537	2599	2738	3031	0	0	0	0	0
Total		185	888000	51506	53398	54961	58122	60240	4402	6017	6653	6823	6,096

^a^Each consulting room works two shifts and provides healthcare to approximately 4800 affiliates

### Study variables

The outcome of interest was glycemic control (yes/no, based either directly on HbA1c levels < 7% or inferred from three consecutive measurements of fasting glucose ≤130 mg/dl levels at the end of each year). The study covariates were: patient sex, age, anthropometric data and nutritional status, history of glycemic control in the year before attending DIABETIMSS, presence, and the number of chronic diabetic complications (diabetic nephropathy, diabetic retinopathy, peripheral neuropathy and peripheral vascular disease) and other comorbidities, such as cardio-vascular diseases (Additional file 1: Table S1). We also explored the following indicators of the quality of the process of care [8]: 1. At least one measurement of HbA1c; 2. Comprehensive foot evaluation; 3. Referral to the ophthalmologist to screen for diabetic retinopathy. 4. Nutritional counseling. 5. Overweight/obese patients receiving metformin, unless contraindicated; 6. Patients with hypertension receiving inhibitors of angiotensin-converting enzyme or angiotensin-receptor blocker, unless contraindicated; 7. Patients aged > 40 years with one or more of the following risk factors for cardiovascular diseases: smoking, hypertension, dyslipidemia, receiving 75–150 mg/day of acetylsalicylic acid unless contraindicated.

### Construction of the analytical database

A structured query language was used to extract the information from the original databases and create the analytical database. The non-plausible values were predefined for the following variables: blood pressure (systolic blood pressure < 50 or > 250 mmHg and diastolic blood pressure < 40 or > 200 mmHg), height (< 130 or > 250 cm), weight (< 30 or > 200 kg), HbA1c (< 3.0) and fasting plasma glucose (< 37 mg/dl). The analysis excluded all non-plausible values that varied among variables from 0.5 to 1.5%. SAS statistical package (V9.2) was used to construct the study variables from the extracted data.

### Statistical analysis

We used relatively new machine learning-based estimators of our intervention impacts of interest (Further details of the definition of the parameters of interest, the estimators and methods for robust inference are in the Supplemental Materials file). The purpose of using such methodology was to create an estimation scheme that avoided unnecessary parametric assumptions, where model selection could be automated towards our goals of interest and would return robust statistical inference.

The data were assumed to be derived on independent individuals with repeated observations (up to 5 depending on enrollment and drop-out). For the treatment impacts, we estimated the average treatment effect (ATE) which can be thought of as a nonparametrically adjusted mean difference in patients in and out of the DIABETIMSS program [21]. We estimated the association parameter separately by each clinic, but we averaged over the repeated years of the study. We evaluated the ATE’s by using a targeted machine learning or in shorthand, Targeted Learning (TL) [18]. To use such an approach, one must estimate both an outcome prediction model and an intervention (DIABETIMSS) model. To do so in an automated and flexible manner, we used a TL approach [18] developed for the programming language R [22]. Internal to this algorithm are initial fits to the distribution (before a targeting step), and that was done using an ensemble machine learning approach [18, 23]. This approach avoids the pitfalls of overly reliance on a single prediction algorithm, allowing for good fits regardless of whether the true model is complex or relatively smooth and straightforward. Besides clinic-specific estimates of the impact of the program, we also created estimates pooled over the intervention clinics to derive average estimate impacts. For all our estimates, we also reported those based upon unadjusted analyses, comparing the proportion of glucose for subject observations both in and out of the program. Also, we reported estimates where we used standard multivariate logistic regression for comparison.

We only had significant missing information on the outcome (62% of observations were missing) and performed complete case analysis assuming the data were missing at random [24, 25]. That is, we assumed there were no other (outcome) predictive covariates available to explain missingness beyond what we used in our models; this means that the conditional regression estimates assume the data are missing at random. We performed standard principal component analysis to compare the covariates for patients with and without missing outcome, and another standard principal component analysis to ensure that clinics had a similar distribution of predictor variables among their populations.

Beyond the analyses of overall intervention impacts, we also attempted to identify patient sub-groups in whom the program had the most significant intervention impacts. We did so by using the machine learning algorithms to predict treatment impact on each of the subject observations in clinics without DIABETIMSS program. Then, we used regression tree, specifically the *rpart* function in R [26] where the outcome was the estimated (predicted) treatment impact, and the covariates were covariates for which we adjusted in the primary analyses of DIABETIMSS impact on glucose control. This method of finding groups with differential treatment impacts can be considered as a tool of precision medicine, and widely used in literature [27, 28].

To explore the greater robustness of the TL approach relative to standard biomedical (epidemiological) regression analyses, we conducted a set of simulations, and compared the performance of the estimates and the confidence intervals of competing methods. Details of the simulations can be found in the Supplemental Materials file.

For sensitivity analysis, we adjusted for the process-of-care indicators in addition to the original adjustment variables, to see if overall associations were importantly different. Thus, in addition to duplicating the analyses, we also look at the distribution of the estimated propensity score.

## Results

The study included up to 78,894 T2D patients that had at least one medical consultation at an FMC during the years analyzed (2012–2016). During this period, 37,767 patients were referred to and attended the DIABETIMSS program at least once (Table 1).

The analysis of simple unadjusted mean differences found a more significant proportion of patients who achieved glycemic control in the DAIBETIMSS program versus not (30 versus 24%). The recent history of glycemic control was a strong predictor of current glycemic control: 61% of patients that had glycemic control during the last year had control in the next year, where only 18% of patients that had lack of control in the previous year, achieved control the following year (*p* < 0.01). There was a significant positive association of age and the glycemic control, but the missing observations drove it. No anthropometric nor nutrition-related variables were related to glycemic control. Those that had multiple risk factors had unexpectedly similar glycemic control as those with no risk factors (24% versus 20%). There was a trend of less glycemic control among those patients with more complications related to diabetes in subjects (24% with no complications, 21% among those with > 1 complication) (Table 2).

**Table 2:** Distribution of glycemic control indicator among predictors, pooled over years and clinics

Variables	HbA1c > =7%	HbA1c < 7%	Missing	Adjusted *p*-value
Referred to DIABETIMSS, n (prop.)				< 0.001
No	63284 (0.50)	31225 (0.24)	33258 (0.26)	
Yes	16254 (0.54)	8940 (0.30)	4797 (0.16)	
Missing	65391 (0.21)	23556 (0.08)	224410 (0.72)	
Previous glycemic control, n (prop.)				<0.001
No	69031 (0.60)	15161 (0.13)	30918 (0.27)	
Yes	12707 (0.26)	19724 (0.41)	15628 (0.33)	
Missing	63191 (0.21)	28836 (0.09)	215919 (0.70)	
Age, n (prop.)				< 0.001
[0,53)	39172 (0.55)	12795 (0.18)	19186 (0.27)	
[53,62)	37784 (0.53)	14612 (0.21)	18642 (0.26)	
[62,71)	38587 (0.53)	18023 (0.25)	16378 (0.22)	
[71, 116]	29386 (0.47)	18291 (0.29)	15395 (0.24)	
Missing	0 (0.00)	0 (0.00)	192864 (1.00)	
Nutrition status at the beginning of the year, n (prop.)				0.646
Underweight	462 (0.44)	190 (0.18)	409 (0.39)	
Normal weight	24399 (0.51)	10454 (0.22)	13326 (0.28)	
Overweight	59249 (0.52)	25584 (0.23)	28164 (0.25)	
Obesity	60609 (0.53)	27360 (0.24)	27228 (0.24)	
Missing	210 (0.00)	133 (0.00)	193338 (1.00)	
Sex, n (prop.)				0.004
Female	86565 (0.52)	38609 (0.23)	40071 (0.24)	
Male	58364 (0.52)	25112 (0.22)	29530 (0.26)	
Missing	0 (0.00)	0 (0.00)	192864 (1.00)	
BMI at the beginning of the year (kg/m^2^), n (prop.)				0.901
[11.2, 26.0)	36448 (0.51)	15636 (0.22)	19581 (0.27)	
[26.0, 28.9)	36040 (0.53)	15495 (0.23)	16969 (0.25)	
[28.9, 32.4)	36242 (0.53)	15979 (0.23)	16096 (0.24)	
[32.4, 85.4]	35989 (0.52)	16478 (0.24)	16481 (0.24)	
Missing	210 (0.00)	133 (0.00)	193338 (1.00)	
Height at the beginning of the year (m), n (prop.)				0.003
[1.30, 1.50)	37877 (0.54)	16438 (0.23)	16342 (0.23)	
[1.50, 1.57)	39267 (0.53)	17393 (0.23)	17526 (0.24)	
[1.57, 1.64)	33231 (0.52)	14773 (0.23)	16363 (0.25)	
[1.64, 2.10]	34344 (0.50)	14984 (0.22)	18896 (0.28)	
Missing	210 (0.00)	133 (0.00)	193338 (1.00)	
Weight at the beginning of the year (kg), n (prop.)				< 0.001
[30, 63)	37150 (0.52)	15744 (0.22)	18099 (0.25)	[30, 63)
[63, 72)	36771 (0.52)	16188 (0.23)	17106 (0.24)	[63, 72)
[72, 82)	35912 (0.53)	15694 (0.23)	16594 (0.24)	[72, 82)
[82, 198]	34886 (0.51)	15962 (0.23)	17328 (0.25)	[82, 198]
Missing	210 (0.00)	133 (0.00)	193338 (1.00)	
Obesity, n (prop.)				0.247
No	24861 (0.50)	10644 (0.22)	13735 (0.28)	
Yes	119858 (0.53)	52944 (0.23)	55392 (0.24)	
Missing	210 (0.00)	133 (0.00)	193338 (1.00)	
Patients with Risk Factors (smoking, hypertension, dyslipidemia), n (prop.)				0.022
No	24358 (0.50)	9576 (0.20)	14853 (0.30)	
Yes	120571 (0.53)	54145 (0.24)	54748 (0.24)	
Missing	0 (0.00)	0 (0.00)	192864 (1.00)	
Smoking Habit, n (prop.)				< 0.001
No	141903 (0.52)	62119 (0.23)	68196 (0.25)	
Yes	3026 (0.50)	1602 (0.27)	1405 (0.23)	
Missing	0 (0.00)	0 (0.00)	192864 (1.00)	
Type of insurance, n (prop.)				0.027
Others	74686 (0.53)	31175 (0.22)	36123 (0.25)	
Parents insured/Retired	70243 (0.52)	32546 (0.24)	33478 (0.25)	
Missing	0 (0.00)	0 (0.00)	192864 (1.00)	
Year, n (prop.)				<0.001
2012	28445 (0.30)	11297 (0.12)	54481 (0.58)	
2013	27127 (0.29)	10916 (0.12)	56180 (0.60)	
2014	29070 (0.31)	12264 (0.13)	52889 (0.56)	
2015	30468 (0.32)	13582 (0.14)	50173 (0.53)	
2016	29819 (0.32)	15662 (0.17)	48742 (0.52)	
Total number of diabetes complications, n (prop.)				<0.001
0	77522 (0.50)	36812 (0.24)	40760 (0.26)	
1	45655 (0.54)	19015 (0.22)	20236 (0.24)	
>1	21752 (0.57)	7894 (0.21)	8605 22)	

The adjusted p-value is derived by tting a generalized estimating equations (GEE) with all the predictors, adjusting for patient ID. Then we did analysis of ‘Wald statistic’ with binomial model and logit link to obtain the p-value. Specifically, the R function is: t = geeglm (formula = indic10 curr diabetimss + edad + sexo + tipo pac + anttab + pesoini + tallaini + imcIni + EdoNutricioIni + facriesg + tot enfcrondiab + SobObes + indic10 prev + year, family = binomial (link = “logit”), data = all complete, id = a l, corstr = “exchangable”, std.err = “san.se” anova (t)

**Fig. 1:**
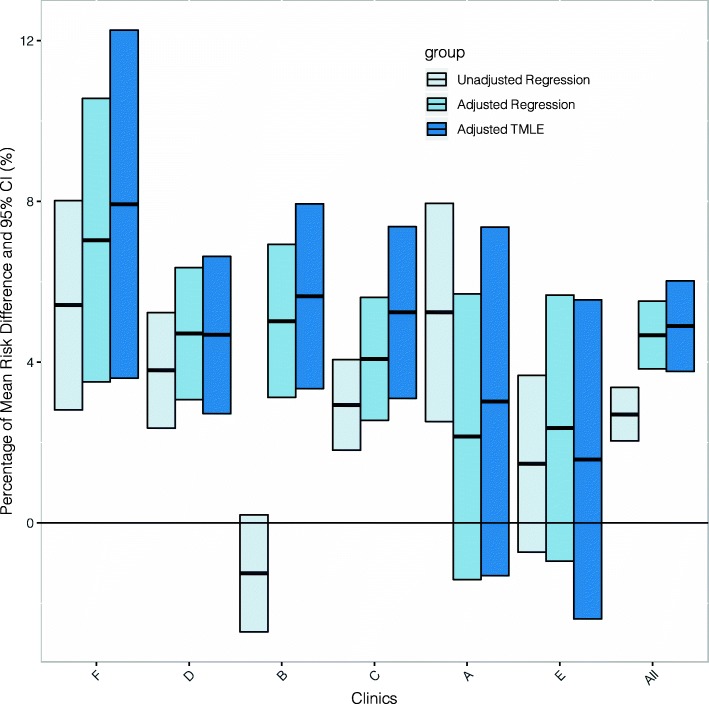
Targeted Learning adjusted associations of DIABETIMSS and glucose control (estimated difference in the percentage of those with HbA1c in two groups) for all DIABETIMSS clinics and all clinics combined (the “All”)

The estimation of the impact of the program revealed that comparing the TMLE results across clinics and pooled (“All” clinics) results, there was a fair amount of variability in the treatment impact; ranging from 2 to 8% improvement in glycemic control, with an overall (pooled) estimate of 5% improvement. Comparing the unadjusted to the two adjusted estimates (standard regression and machine-learning adjusted TMLE) showed strong evidence of confounding by the measured factors. For most clinics, the adjusted estimates were generally more significant than the unadjusted (Fig. 1 and Table 3).

**Table 3:** Associations of DIABETIMSS program and glycemic control indicator by clinic and pooled over all clinics

Clinic	DIABETIMSS	n	Unadjusted logistic regression	Adjusted logistic regression	TMLE
			HbA1c < 7% (95% CI)	HbA1c < 7% (95% CI)	HbA1c < 7% (95% CI)
A	No	4778	0.3599 (0.3517, 0.3680)	0.3692 (0.3574, 0.3810)	0.3694 (0.3577, 0.3812)
	Yes	573	0.4122 (0.3864, 0.4381)	0.3907 (0.3564, 0.4249)	0.3997 (0.3665, 0.4329)
	RD		0.0524 (0.0252, 0.0795)	0.0215 (−0.0141, 0.0570)	0.0302 (−0.0131, 0.0736)
B	No	6335	0.4027 (0.3950, 0.4103)	0.4028 (0.3918, 0.4138)	0.4034 (0.3926, 0.4143)
	Yes	2324	0.3901 (0.3777, 0.4025)	0.4530 (0.4368, 0.4693)	0.4598 (0.4444, 0.4752)
	RD		-0.0125 (−0.0271, 0.0020)	0.0502 (0.0312, 0.0693)	0.0564 (0.0334, 0.0794)
C	No	11535	0.3419 (0.3369, 0.3470)	0.3285 (0.3222, 0.3349)	0.3291 (0.3228, 0.3354)
	Yes	3269	0.3713 (0.3612, 0.3814)	0.3694 (0.3554, 0.3833)	0.3815 (0.3673, 0.3957)
	RD		0.0293 (0.0181, 0.0406)	0.0408 (0.0255, 0.0561)	0.0524 (0.0310, 0.0737)
D	No	3500	0.3067 (0.2982, 0.3151)	0.2911 (0.2801, 0.3022)	0.2917 (0.2809, 0.3026)
	Yes	2208	0.3446 (0.3330, 0.3563)	0.3382 (0.3252, 0.3513)	0.3385 (0.3257, 0.3513)
	RD		0.0380 (0.0236, 0.0523)	0.0471 (0.0307, 0.0635)	0.0468 (0.0272, 0.0663)
E	No	1050	0.1570 (0.1483, 0.1657)	0.1624 (0.1503, 0.1746)	0.1623 (0.1501, 0.1744)
	Yes	229	0.1717 (0.1514, 0.1919)	0.1860 (0.1551, 0.2169)	0.1781 (0.1493, 0.2069)
	RD		0.0147 (−0.0073, 0.0367)	0.0236 (−0.0095, 0.0567)	0.0158 (−0.0239, 0.0555)
F	No	1160	0.2049 (0.1944, 0.2154)	0.2370 (0.2219, 0.2520)	0.2376 (0.2226, 0.2527)
	Yes	337	0.2590 (0.2352, 0.2828)	0.3073 (0.2753, 0.3393)	0.3169 (0.2844, 0.3494)
	RD		0.0542 (0.0281, 0.0802)	0.0703 (0.0351, 0.1056)	0.0793 (0.0360, 0.1226)
All	No	28325	0.3278 (0.3247, 0.3309)	0.3225 (0.3184, 0.3266)	0.3227 (0.3185, 0.3268)
	Yes	8940	0.3548 (0.3489, 0.3608)	0.3692 (0.3617, 0.3768)	0.3716 (0.3639, 0.3794)
	RD		0.027 (0.0204, 0.0337)	0.0467 (0.0383, 0.0552)	0.0490 (0.0377, 0.0602)

The estimates displayed in this table represent the proportion of subjects with HbA1c < 7% within each group (DIABETIMSS Yes/No) as well as the difference of these proportions in the two groups. We show three estimators as discussed in text: unadjusted, adjusted within a logistic regression and finally using targeted maximum likelihood estimation (TMLE)

**Fig. 2:**
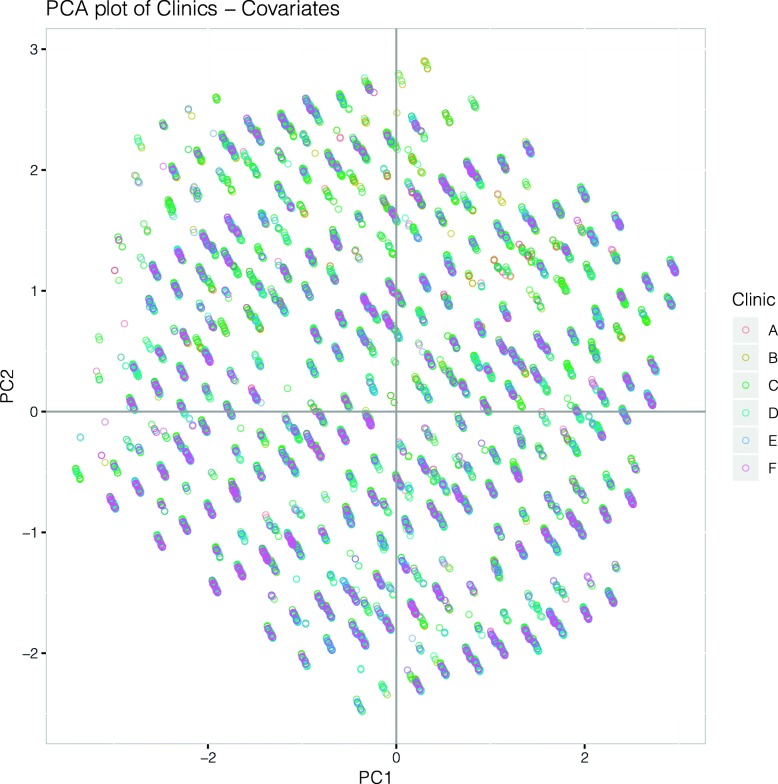
Principal components analysis of DIABETIMSS clinics

To explore whether some clinics had very different distributions of predictors, we performed a standard principal component analysis (PCA) and colored the points on a resulting PCA plot by each clinic (Fig. 2), which shows consistent overlap among clinics. This finding suggests that there were no dramatic differences in covariate distributions among the 6 DIABETIMSS program clinics. The results of running logistic regression stratified by clinic also showed a relatively consistent associations of covariates across clinics (Additional file 1: Figure S3).

**Fig. 3:**
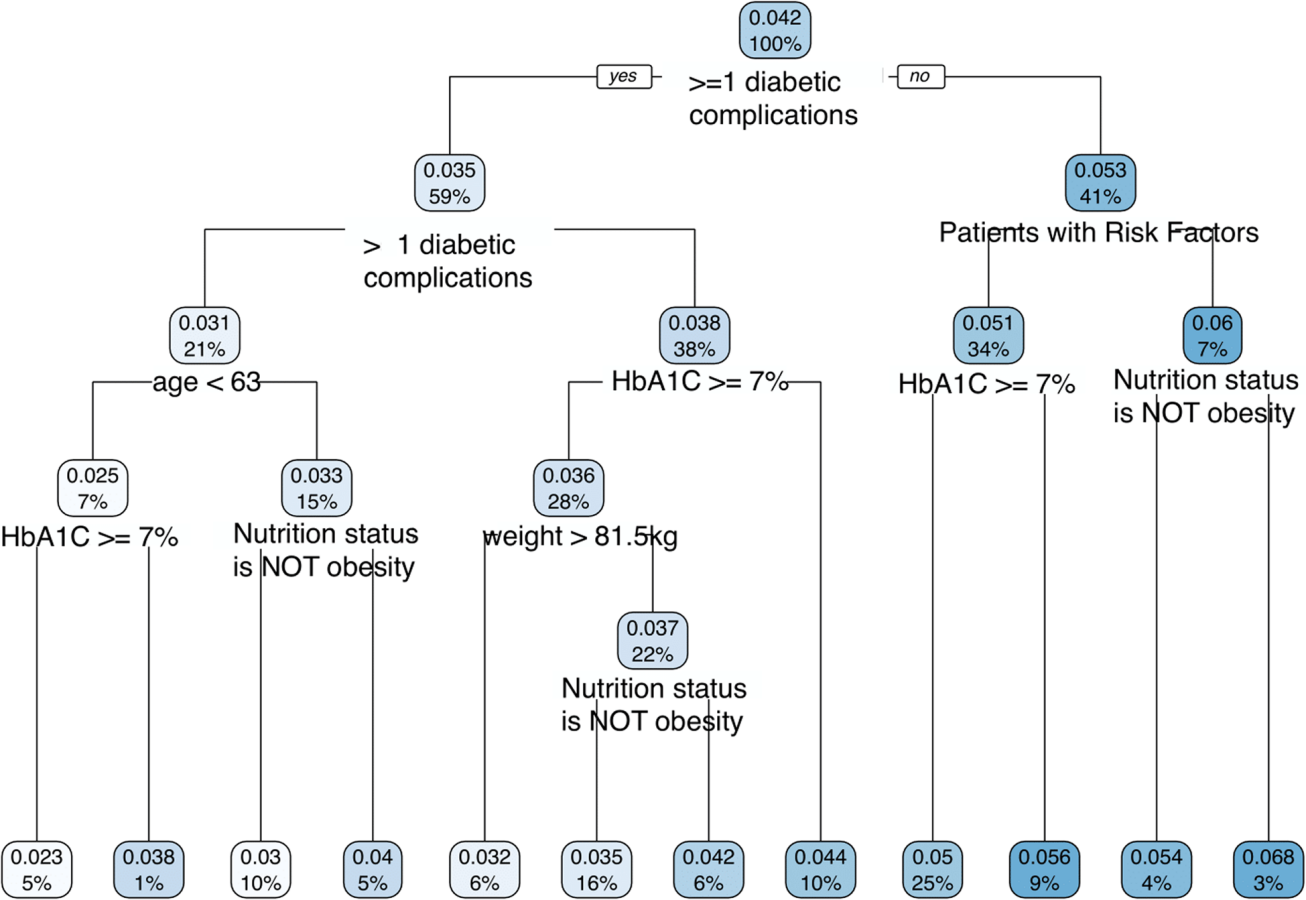
Tree diagram showing the predicted treatment effect subgroups in control clinics

The distribution of estimated individual treatment effect (Additional file 1: Figure S4) showed a relatively notable extent of heterogeneity. To explain this heterogeneity, we performed regression tree on the blip-function transformed data (*Y*^∗^) to explore the factors most responsible for differences in the treatment impact. Regression tree is a simple form of histogram regression based on binary splits on covariates. It results in distinct nodes (representing sub-populations) that “best” characterize the variability seen in the outcome (in our case, the blip function). We found that the terminal nodes (the smallest subgroups) vary in their treatment impact from relatively low (2.6% in the leftmost node) to modestly larger than the average treatment effect (6.4%). If we examine the variables that define these splits, if there is a general message, it is that those with fewer existing complications of diabetes appear to have a more significant benefit from the program than those with more complications (Fig. 3). This result is not surprising as the magnitude of the reversal of the disease progression is more meaningful and harder to achieve among this subset. However, one sees no distinct sub-populations where either the program is universally effective or vice versa. Thus, for any group, using the average impact estimated (around 5% improvement) is not an unrealistic estimate.

**Fig. 4:**
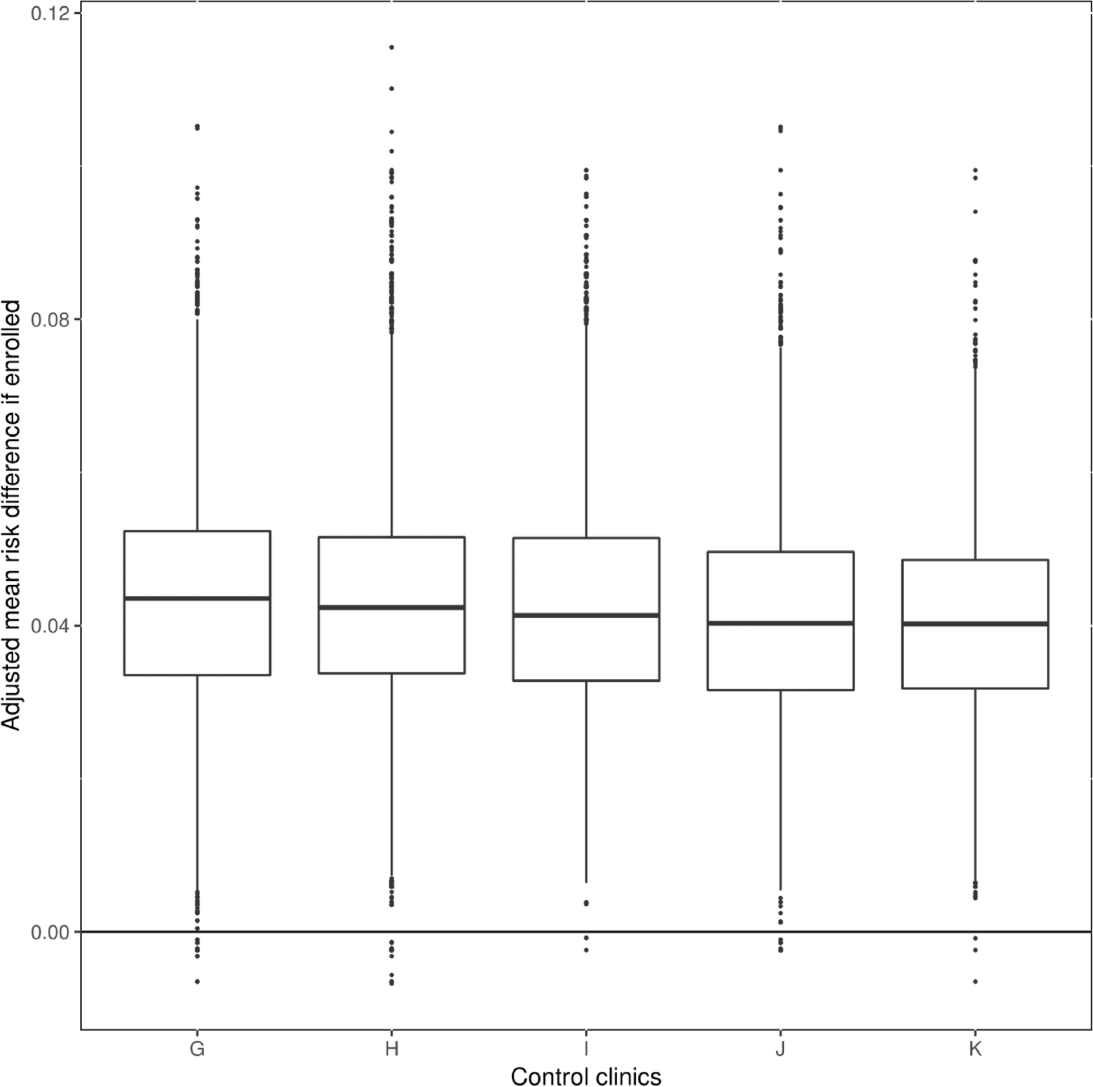
Boxplot of predicted impact of implementing DIABETIMSS program in control clinics

We also predicted the impact on a patient by patient basis for the conventional model clinics. We found that the predicted impact is quite like what was observed empirically in the DIABETIMSS clinics, that is, there is some variation, but one would expect about a 5% improvement in glycemic control (on average) if the program were implemented in these clinics (Fig. 4).

**Fig. 5:**
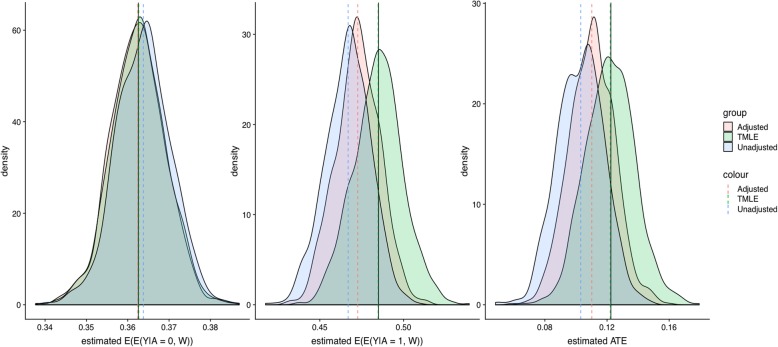
Distribution of model estimation using original data parameters

**Fig. 6:**
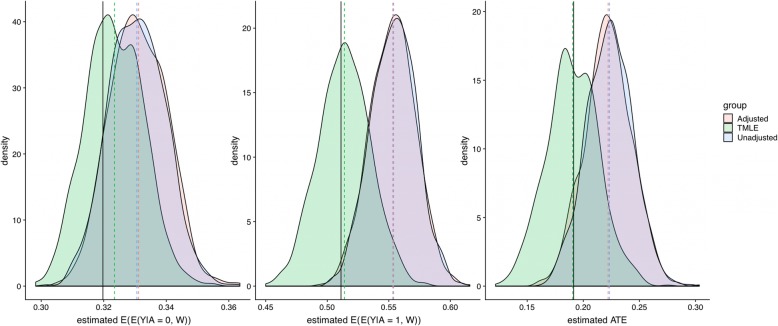
Distribution of model estimation using more variant data parameters

The simulations revealed that the performance of the TMLE estimator is far superior to the simpler estimators (Figs. 5 and 6). Mainly, TMLE still works in cases when the parametric approaches fail to pick up the confounding and result in poor approximations for the true prediction model. More details can be found in the Supplemental Materials file.

**Fig. 7:**
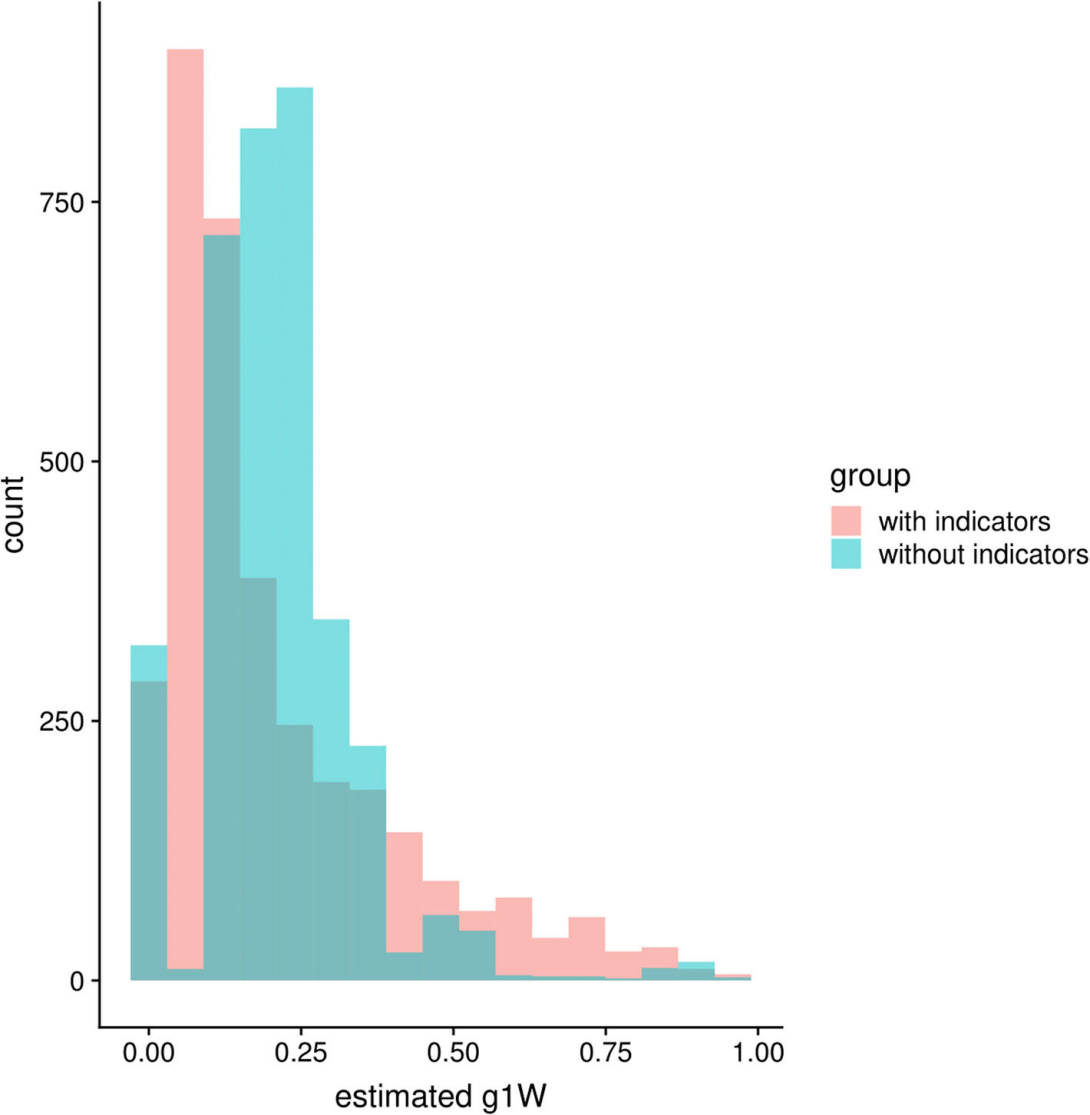
Distribution of estimated propensity scores, g(W) both including and excluding the process-of-care indicators

The sensitivity analysis that included process-of-care indicators as confounders showed more variable results, but the overall estimate averaged across all clinics did not change substantially (Additional file 1: Figure S1). Thus, adjustment by these indicators did not change the main conclusions of the analysis. One can see that the distribution of propensity scores (Fig. 7) has a larger proportion of the distribution at very low values (near 0) when the process-of-care indicators are included in the adjustment set. This result indicates other variable importance results (not included but available upon request) that suggests a weak association of these indicators with the outcome, but a strong correlation with the program, again suggesting they are problematic as confounders for our outcome (HbA1c indicator).

## Discussion

The study provides evidence on the positive effect of DIABETIMSS program (pooled estimate of a 5% of improvement in glycemic control) and shows the potential and challenges in using routine observational patient data and machine learning methods to evaluate the performance of health interventions within complex healthcare institutions to inform decision-makers.

DIABETIMSS was implemented to improve diabetes care and health outcomes by addressing three critical elements of the Chronic Care Model (CCM): 1) re-design of the delivery system through multidisciplinary teams, 2) decision support through evidence-based clinical guidelines, and 3) counseling and empowering of patients on self-management. Multiple clinical trials in different countries have tested these three elements, showing positive effects on the improvement of the processes of care and patients’ outcomes [29, 30]. CCM has been increasingly advocated for effective management and control of NCDs within primary care [31]. Results from randomized controlled trials that have tested CCMs in primary care contexts in Europe show that compared to usual diabetes care, more patients reached treatment targets for blood pressure, and levels of blood sugar and cholesterol [32]. Experiences with CCMs in 8 Caribbean countries show improvements in baseline to follow up measures of blood glucose control and increases in the proportion of patients receiving a preventive practice or meeting quality-of-care indicators [33].

DIABETIMSS evaluation results are consistent with other CCMs interventions, revealing a small but essential impact of this program with an overall pooled estimate of 5% improvement in glycemic control of T2D patients. Nonetheless, this slight increase in the percentage of T2D patients who achieved glycemic control call for further research, as IMSS’ decision-makers require additional evidence to ascertain whether DIABETIMSS provide the interventions of the CCM optimally in compliance with evidence-based guidelines to assure high-quality care and better health outcomes [31, 34]. The evidence suggests that more significant benefits could be obtained through combining all six elements of the CCM that means incorporating the organizational changes that focus on creating a culture and mechanisms that promote safe, high-quality care, including the introduction of strategies to facilitate changes, and management of errors and quality control problems [30]. Another critical element of the CCM is the availability of timely and accurate health information systems to ensure program accountability and provide information for future improvement efforts [31].

The outcome variable of this study was HbA1C < 7%. Since 2000, this goal is recommended by the IMSS diabetes clinical guidelines, independently of patient age. However, since 2016, American Diabetes Association (ADA), highlighted that HbA1C measurement may have limitations primarily in older adults who have medical conditions that increase red blood cell turnover (e.g., hemodialysis, recent blood loss or transfusion, or erythropoietin therapy), which can falsely increase or decrease A1C. Therefore, for adults ≥65 years of age ADA recommends specific glycemic control goals of HbA1C < 7.5% for healthy older adults with few coexisting chronic illnesses and HbA1C < 8.0% or < 8.5% for older adults with multiple coexisting chronic illnesses or instrumental impairments or cognitive impairment [35]. If we apply the ADA recommendation to our study, this could probably increase the effect of the DIABETIMSS on glycemic control of older patients; yet, further analysis is recommended to support this hypothesis.

To date, diabetes research that used machine learning methods, was focused primarily on biomarker identification, prediction of diagnosis and diabetes complications, with low emphasis on evaluation of healthcare programs [36]. Our study is one of the pioneers to evaluate the performance of an ongoing health program using machine learning methods and routine observational patient data to inform decision-makers. The study showed both the potential and challenges in using detailed observational patient data to evaluate the performance of a healthcare program. Though the estimates from standard regression were not radically different from those based upon less biased, machine learning methods, they do show enough difference to be important, mainly when the impacts apply to so many patients. The simulations show that the more complex targeted learning estimator does not harm performance when a more straightforward model provides an adequate approximation. However, usually, it is difficult to know at the beginning of the study whether standard methods will suffice, although, using such methods could increase the risk of misleading conclusions.

The present study shows the merits of using targeted learning approaches to evaluate the average performance of the intervention and explore its heterogeneity across different clinics. The analyses based on the distribution of patient characteristics also provide information regarding which clinics are most likely to benefit from future expansion of DIABETIMSS. The information provided could be the basis of informed cost-benefit analyses of DIABETIMSS or other programs.

Finally, the study allowed for creating the basis for an analytical framework that can be applied across complex health systems for evaluating programs/treatments using sophisticated machine learning technology but with simple interfaces for non-technical users.

The limitations of the analysis are related to the deficiencies of the available data. First, one of the limitations is related to the inclusion in the analysis of all patients with at least one visit to DIABETIMSS program during the calendar year. We based this decision on the fact that according to the DIABETIMSS internal handbook the first visit to DIABETIMSS should include individual patient consultation about self-care with the medical doctor and dietitian and group consultation with the nurse and social worker. It is expected that during this first visit, the patient will receive valuable information and motivation to his/her self-care and continue attending to the group education sessions on self-care. The average duration of the first patient consultation is 3 h. Also, this decision can be explained by the fact that currently, IMSS lacks information on the number of visits and group educational sessions that each patient had in DIABETIMSS during one calendar year that is the usual time of DIABETIMSS exposure. The available information only includes the first consultation with the DIABETIMSS multidisciplinary team of health professionals. This situation impairs to identify the extent of exposure, particularly the optimal number of visits and group educational sessions to improve patient glycemic control. However, DIABETIMSS aim for a patient is to attend to 12 group educational sessions during one calendar year. IMSS could benefit from collecting routine information on the number of individual consultations and group sessions to evaluate the effect of the extent of exposure.

Second, the data had significant missing values, particularly for the outcome, making extrapolation of the results on those non-missing observations more problematic for the entire population. We assumed missing at random (MAR). This is the weakest identifiability assumption we could take and still estimate the impact of the program. MAR is also not identifiable empirically, so it is always non-testable. We found no observable difference in the principal component analysis of the covariates for patients with and without missing outcome (Additional file 1: Figure S2). In this case, we had a predictive set of covariates to predict the outcome (and are not missing) and using Super Learner insures that all information about the outcome contained in them is used. This is typically better than most handing of missing data (such as parametric imputation or inverse weighting).

## Conclusions

Machine learning methods that use routine observational patient data is useful to evaluate the performance of an ongoing health program to inform decision-makers. Beyond the specific application to DIABETIMSS, the combination of methods and data suggest this type of study is valuable for evaluating programs and treatments within complex health care systems.

## Supplementary information

**Additional file 1:** **Table S1.** Description of covariates. **Figure S1.** Associations of DIABETIMSS and glucose control adjusting for process-of-care variables (estimated difference in the percentage of those with HbA1c in two groups) **Figure S2.** Principal components analysis of covariates for patients with and without missing outcome. **Figure S3.** Comparison of associations of covariates and outcome by clinic. **Figure S4.** Distribution of DIABETIMSS treatment impacts among subjects in DIABETIMSS clinics.

## Data Availability

The datasets analysed during the current study are not publicly available due to the IMSS internal rules but are available from Alan Hubbard (hubbard@berkeley.edu) on reasonable request.

## Abbreviations

ATE: Average treatment effect
FMCs: Family medicine clinics
IMSS: Mexican Institute of Social Security
T2D: Type 2 diabetes.
TL: Targeted Learning
