# Supplementary material for: Application of machine learning methodology to assess the performance of DIABETIMSS program for patients with type 2 diabetes in family medicine clinics in Mexico
Source: BMC Med Inform Decis Mak. 2019 Nov 12;19:221. doi: 10.1186/s12911-019-0950-5 (PMC6852791; doi:10.1186/s12911-019-0950-5)
Supplement: Supplementary file 1 — Additional file 1: Table S1. Description of covariates. Figure S1. Associations of DIABETIMSS and glucose control adjusting for process-of-care variables (estimated difference in the percentage of those with HbA1c in two groups) Figure S2. Principal components analysis of covariates for patients with and without missing outcome. Figure S3. Comparison of associations of covariates and outcome by clinic. Figure S4. Distribution of DIABETIMSS treatment impacts among subjects in DIABETIMSS clinics. [file 12911_2019_950_MOESM1_ESM.docx]

# Supplemental Materials

## **Statistical Methods**

***Data Structure***

In order to understand the estimation procedure, one must have some notation to describe the data structure on how our estimates average over different units (years, people, clinics). We assumed the data were independent individuals, *i = 1,…,m*, with repeated observations, *j = 1,…, n_i_*, that is, we allowed for some subjects to have fewer than the total possible number of times, 5. Then the data on each person can be represented by:

$O_{i}\equiv(R_{ij}, T_{ij}, W_{ij}, A_{ij}, Y_{ij}, i=1,\cdots,n_{i})$,

where *R_ij_* is the specific clinic, *T_ij_* is the year of the program and measurement of outcome, *A_ij_* is the indicator of the DIABETIMSS program (1 = yes), *W_ij_* is the set of confounders that can include measurements made in past years, and *Y_ij_* is the indicator of glucose control. In this analysis, we treated the data like a serial cross-sectional study, so defined the observed data for an individual at time *T_ij_ = t*. In this case, *P_0_(O(T_ij_=t))* is the joint data-generating distribution of the data of observations made at time t.

***Parameter of interest***

We estimated the association parameter based upon causal inference, separately by clinic, *R_ij_ = r*, but we averaged the impact over the years (*T_ij_ = t*) of the study. We defined the yearly parameter of interest as:

$$ATE\left( r \right)\equiv E\left[ E\left( Y_{1}-Y_{0} | T_{ij}=t,R_{ij}=r \right) \right],$$

where the inner conditional expectation, *E(Y_1_-Y_0_ | T_ij_ = t; R_ij_ = r)* is the conditional (on year and clinic) average treatment effect (ATE), where *Y_1_* is the so-called counterfactual outcome if the patient, possibly contrary to fact, had the intervention (*Y_0_* is control outcome). Our parameter was defined as the mean of the annual association over the years of the program (T = 2012,…,2016).

Of course, one cannot estimate this directly, given there are no measured outcomes under both interventions (DIABETIMSS and control) for the same patient in the same year, so one estimates this quantity by asserting certain assumptions to derive an estimand (a function of the actual data-generating distribution) or:

$$\Psi\left( P \right)\left( r \right)=E\left\{ E\left( Y_{1}- Y_{0} | T_{ij}=t,R_{ij}=r \right) \right\}= \Psi\left( P_{0} \right)\left( r \right)$$

$=E\left\{ E\left[ E\left( Y_{ij} | A_{ij}=1, T_{ij}=t, W_{ij}=w_{ij}, R_{ij}=r \right)- E\left( Y_{ij} | A_{ij}=0, T_{ij}=t, W_{ij}=w_{ij}, R_{ij}=r \right) \right] \right\}$(1)

which represents three nested averages under identification assumptions: the inner conditional mean given *W_ij_=w_ij_, R_ij_=r*, and *T_ij_=t*, the next going out is over *W_ij_ given T_ij_=t, R_ij_=r* and finally the outer expectation is over *T_ij_ given R_ij_=r*. In orders, for a fixed clinic, $r$ and fixed time $t$, one gets the difference in predicted values when a patient is in versus out of the DIABETIMSS program, then one averages these differences over all times within the clinic to derive the parameter. Finally, one can take the weighted mean over all the clinics to define an overall pooled estimator. We estimated the population impact of the DIABETIMSS program, defined by the difference in adjusted means among observations (years) of patients in versus out of the DIABETIMSS program, *only within the clinics that had patients both in and out of the program.* Specifically, we estimated the adjusted means, defined by the average of predictions (based upon different regression approaches) when all observations are assigned to DIABETIMSS, versus the same observations with patients being assigned to control group. In notation, we estimated for each clinic $R=r$:

$$\hat{ATE}\left( r \right)= \frac{1}{n_{r}}\sum_{t=2012}^{2016} \sum_{i=1}^{m} \sum_{j=1}^{n_{i}} I\left( R_{ij}=r \right)*I\left( T_{ij}=t \right)*(\hat{Q}\left( 1, W_{ij}, t, r \right) - \hat{Q}(0, W_{ij}, t, r))$$

where $n_{r}=\sum_{i=1}^{m} \sum_{j=1}^{n_{i}} I(R_{ij}=r)$, or the number of observations in clinic r, $\hat{ATE}(r)$ is the estimated average treatment effect (the name of the parameter), and $\hat{Q}\left( a,w,t,r \right)=\hat{E}(Y|A=a,T=t,W=w,R=r)$ or the result of an estimated regression of $Y$ on $\{A,T,W,R\}$. We averaged the $\hat{ATE}\left( r \right)$ over the $r$ to get the overall pooled average, $\hat{ATE}$. All of our estimators are defined by how we derived $\hat{Q}\left( a,w,t,r \right)$, and we did so 3 different ways based upon: 1) simple unadjusted means in each group, 2) adjusted means, where adjustment was via standard main terms logistic regression and 3) adjusted for covariates using machine-learning-based Targeted Learning methods [van der Laan, M. and Rose, S. (2011). Targeted learning: causal inference for observational and experimental data. Springer.]. For 1) $\hat{Q}\left( a,w,t,r \right)$ is simply the proportion of observations with $Y_{ij}=1$ among all observations in clinic $r$ $(R_{ij} = r)$ within year $(T_{ij} = t)$. For 2), we fitted main terms logistic regression, so:

$$\hat{Q}\left( a,w,t,r \right)=\frac{1}{1+exp\{-\left( \hat{\alpha}_{0,t, r}+\hat{\alpha}_{1,t, r}a+\hat{\beta}_{1,t, r}w_{1}+\hat{\beta}_{2,t, r}w_{2}+\ldots+\hat{\beta}_{p,t, r}w_{p} \right)\}}$$

which is a multivariate logistic regression done separately by clinic and time. For 3) we used an ensemble machine learning method called Super Learning [van der Laan, M. J., Polley, E. C., and Hubbard, A. E. (2007). Super learning. Stat Appl Genet Mol Biol, 6: Article25], augmented with a method based upon targeted maximum likelihood estimation (tmle; Laan, M. J. v. d. and Rubin, D. B. (2006). Targeted maximum likelihood learning. International Journal of Biostatistics, 2(1). Article 11] to insure Gaussian sampling distribution and bias reduction via the addition of a “clever covariate”.

We only had significant missing information on the outcome (62% of observations were missing), thus we performed complete case analysis assuming the data were missing at random [REF: RUBIN, D. (1976). INFERENCE AND MISSING DATA. BIOMETRIKA, 63(3):581–590.]. That is, we assumed there were no other (outcome) predictive covariates available to explain missingness beyond what we used in our models; this means that the conditional regression estimates assume the data are missing at random.

We performed a standard principal components analysis to explore whether some clinics had very different distributions of predictors.

Besides, we identified patient sub-groups in whom the program was working best by performing tree regression on the blip-function transformed data in clinics without DIABETIMSS [REF: Robins, J.M., 2000. Marginal structural models versus structural nested models as tools for causal inference. In *Statistical models in epidemiology, the environment, and clinical trials* (pp. 95-133). Springer, New York, NY.].

***Simulations***

To explore the greater robustness of the TL approach relative to standard biomedical (epidemiological) regression analyses, we conducted a set of simulations. We based the simulations closely upon the actual data, using a specific clinic's data to estimate the data-generating distributions. We used flexible, machine learning methods to estimate both the outcome and treatment models. We then ran simulations based upon this model (can be thought of as a semi-parametric bootstrap) and one where more non-linearity was entered into estimation of the prediction model. We then compared the performance of the estimates and the confidence intervals of competing methods.

The purpose is to show the greater robustness of the Targeted Learning approach to estimation of adjusted associations.

**Results of simulations**

Figure 5 shows the plots of the sampling distribution along with the mean of the 3 estimators and the true mean (black line). In addition, the caption contains specific numbers regarding the performance of the different estimators. The left and middle plots are the estimates of one component of the ATE (adjusted mean when A = 0 and A = 1, respectively). The farthest right is that of the parameter of interest, the ATE. One can see small reduction in bias in the TMLE, versus the standard adjusted and unadjusted. However, even the mean of the unadjusted estimates is close to the true value, and its confidence interval has nearly perfect 95% coverage, so there is very little room for improvement.

This is why we also used an augmented distribution to examine the relative performance when there is potential confounding by measured covariates as well as important non-linearities in the true prediction model. Figure 6 shows of the results of these simulations, along with detailed information on the relative importance. shows the three distributions with their asymptotic mean values, MSE and coverage rate for the 95% confidence interval. Clearly, the performance of the TMLE estimator is far superior to the other two simpler estimators - they fail to pick up the confounding and are poor approximations for the true prediction model.

The message is that, if simpler, more parametric approaches work, so does TMLE (Figure 5). However, TMLE still works in cases where they fail (Figure 6).

**Supplementary table 1: Description of covariates**

| **Variable** | **Type** | **Cathegories** |  |
| --- | --- | --- | --- |
| Age | continuous |  |  |
| Sex | binary | 1) Female 2) Male |  |
| Insuarance status | categorical | 1) Insured; 2) Spouse of insured; 3) Child of insured; 4) Parents insured; 5) Retired |  |
| Smoking habit | binary | 0) No 1) Yes |  |
| Weight at the beginning of the year (kg) | continuous |  |  |
| Height at the beginning of the year (m) | continuous |  |  |
| BMI at the beginning of the year (kg=m^2^) | continuous |  |  |
| Nutrition status at the beginning of the year | categorical | 1) Underweight; 2) Normal weight; 3) Overweight 4) Obesity |  |
| Overweight / Obesity | binary | 0) No 1) Yes |  |
| Total number of diabetic complications | continuous |  |  |
| Indicator 10: Having HbA1C <7% in the last |  |  |  |
| measurement; or in the absence of HBA1 test fasting glucose <= 130mg/dl in the last 3 measurement in previous year | binary | 0) No 1) Yes |  |
| Patients with Risk Factors (smoking; hypertension; dyslipidemia) | binary | 0) No 1) Yes |  |
| record year | categorical | 2012 to 2016 | |
| Process-of-care indicators |  |  | |
| Indicator 1: Referral to the screening for dyslipidemia by measuring total cholesterol in patients without previous dyslipidemia | binary | 0) No 1) Yes | |
| Indicator 2: At least one measurement of HbA1C | binary | 0) No 1) Yes | |
| Indicator 3: Comprehensive foot evaluation | binary | 0) No 1) Yes | |
| Indicator 4: Referral to the ophthalmologist | binary | 0) No 1) Yes | |
| Indicator 5: At least one nutritional counseling provided by the nutrition service | binary | 0) No 1) Yes | |
| Indicator 6: Overweight and obese patients who received metformin unless contraindicated | binary | 0) No 1) Yes | |
| Indicator 7: Patients with hypertension receiving inhibitors of angiotensin converting enzyme (IACE) or angiotensin-receptor blocker unless contraindicated | binary | 0) No 1) Yes | |

Weight, height and BMI are correlated variables, however, the machine learning methods we used will automatically perform variable selection to select the significant ones, so that collinearity among adjustment variables does not hurt the estimator.  In general, if using Super Learner with a set of data-adaptive algorithms, theory predicts that it’s best, when in doubt, to include an adjustment variable (see Oracle Inequality in van der Laan, Mark J., Eric C. Polley, and Alan E. Hubbard. "Super learner." Statistical applications in genetics and molecular biology 6.1 (2007)).

**Supplementary figure 1: Associations of DIABETIMSS and glucose control adjusting for process-of-care variables (estimated difference in the percentage of those with HbA1c in two groups),**

Targeted Learning adjusted associations of DIABETIMSS and glucose control for all DIABETIMSS clinics that includes adjust for process-of-care variables.

**Supplementary figure 2: Principal components analysis of covariates for patients with and without missing outcome.**

**
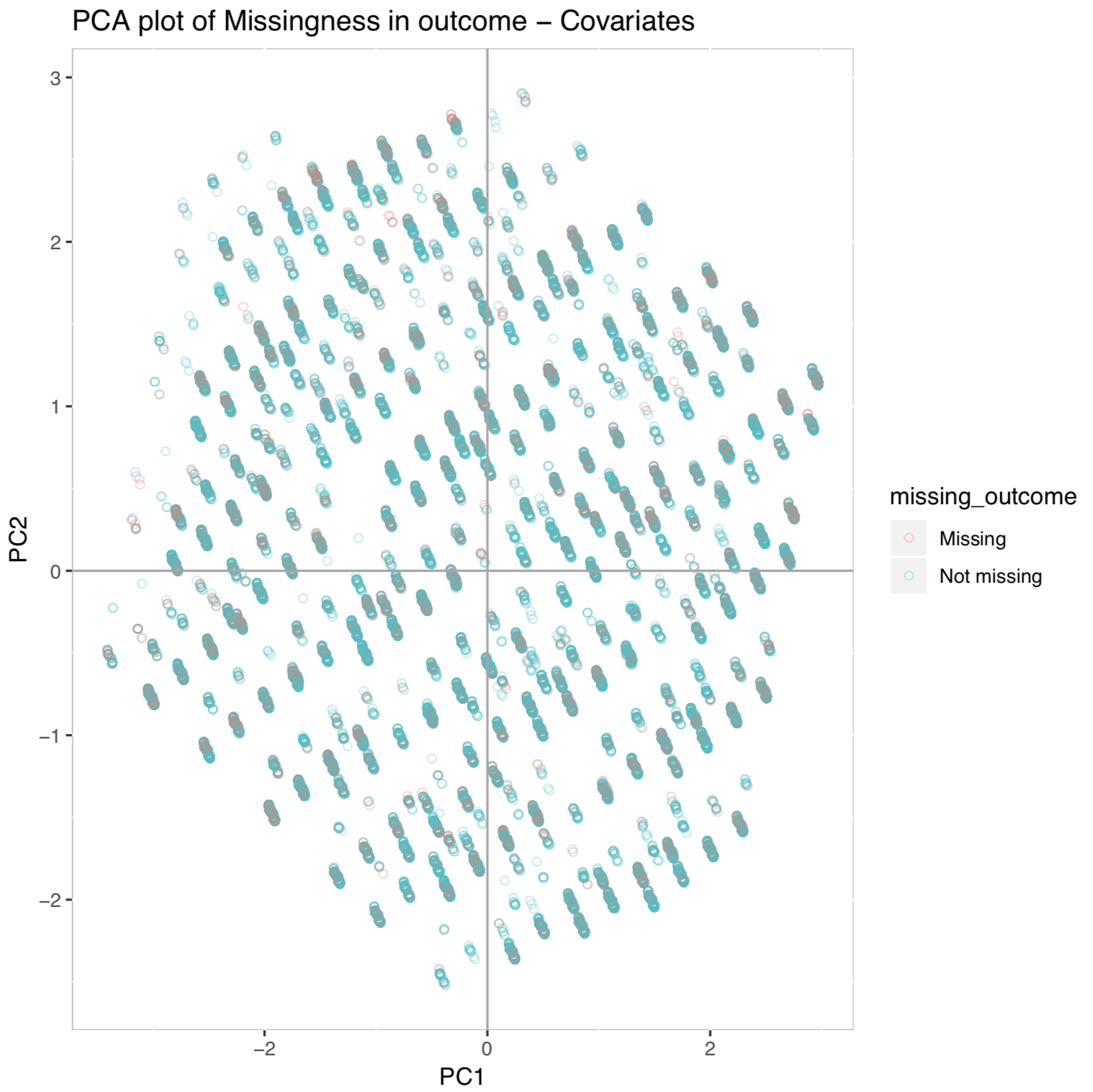
**

**Supplementary figure 3: Comparison of associations of covariates and outcome by clinic.**

The first 2 variables stand for 2 nominal levels of total number of diabetic complications. The 3 levels of total number of diabetic complications are: 0, 1, > 1.

**Supplementary figure 4: Distribution of DIABETIMSS treatment impacts among all subjects in DIABETIMSS clinics.**

**
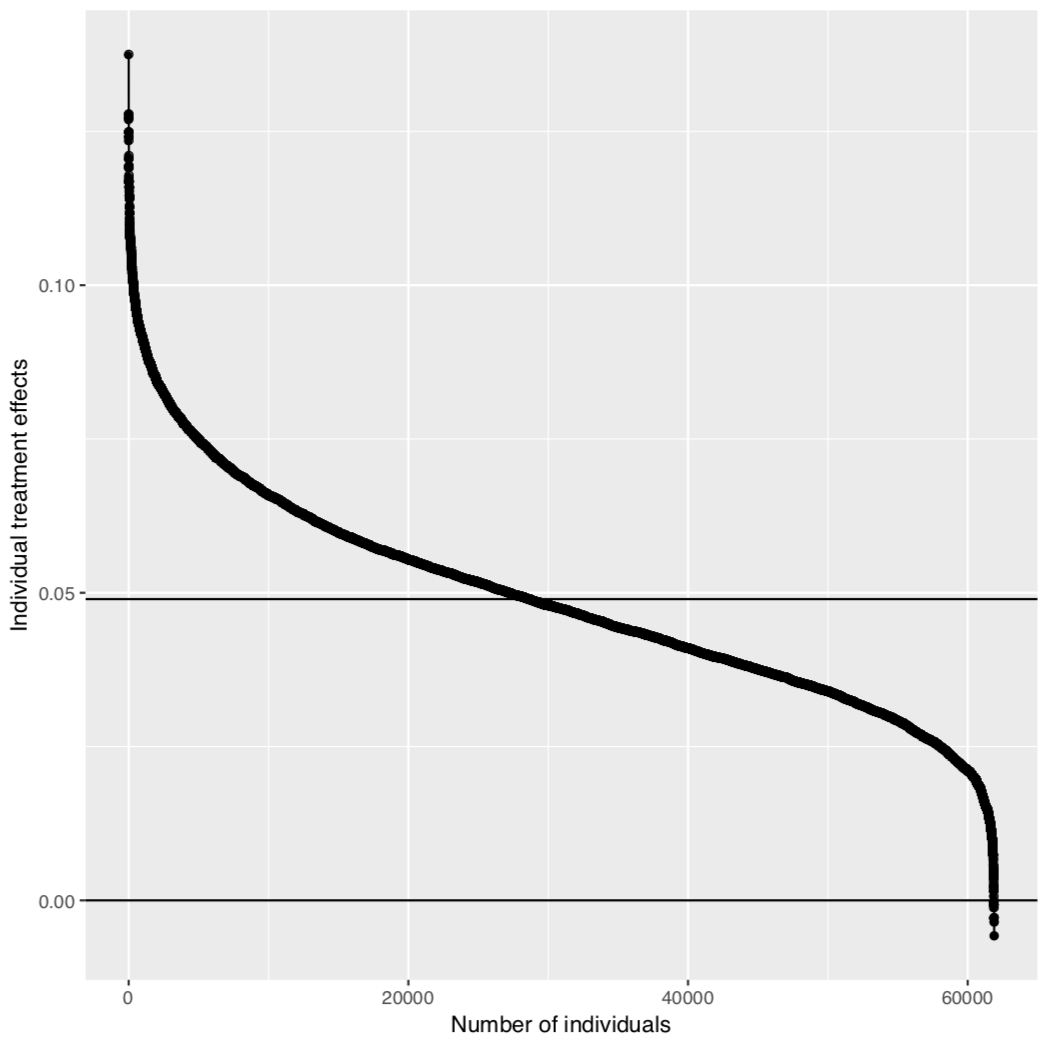
**
